# Order short-term memory is not impaired in dyslexia and does not affect orthographic learning

**DOI:** 10.3389/fnhum.2014.00732

**Published:** 2014-09-23

**Authors:** Eva Staels, Wim Van den Broeck

**Affiliations:** Department of Clinical and Lifespan Psychology, Faculty of Psychology and Educational Sciences, Vrije Universiteit BrusselBrussels, Belgium

**Keywords:** dyslexia, short-term memory, serial order, reading acquisition, orthographic learning, phonological processing

## Abstract

This article reports two studies that investigate short-term memory (STM) deficits in dyslexic children and explores the relationship between STM and reading acquisition. In the first experiment, 36 dyslexic children and 61 control children performed an item STM task and a serial order STM task. The results of this experiment show that dyslexic children do not suffer from a specific serial order STM deficit. In addition, the results demonstrate that phonological processing skills are as closely related to both item STM and serial order STM. However, non-verbal intelligence was more strongly involved in serial order STM than in item STM. In the second experiment, the same two STM tasks were administered and reading acquisition was assessed by measuring orthographic learning in a group of 188 children. The results of this study show that orthographic learning is exclusively related to item STM and not to order STM. It is concluded that serial order STM is not the right place to look for a causal explanation of reading disability, nor for differences in word reading acquisition.

## Introduction

Developmental dyslexia is commonly defined as a disability characterized by low reading achievement and deficiencies in learning to spell and write (Snowling, 2012). Since the beginning of the research into dyslexia, a number of causal hypotheses have been formulated. The most dominant theory attributes the specific problems associated with dyslexia to a phonological processing deficit (for reviews, see Stanovich and Siegel, 1994; Vellutino et al., 2004; Ziegler and Goswami, 2005). However, based on the observations that dyslexic persons often show impairments on a wider variety of cognitive tasks, some researchers believe that the underlying cause of dyslexia should be situated in a more general process. Recently several new causal hypotheses have been formulated. Ahissar and co-authors proposed the anchoring-deficit hypothesis (Ahissar et al., 2006) which suggests that dyslexics have a general difficulty in automatic extraction of stimulus regularities from auditory inputs. Also recently formulated is the visual attention span hypothesis which proposes that difficulties in processing visual elements simultaneously is at least one cause of dyslexia (Bosse et al., 2007), and according to the visual crowding hypothesis, dyslexics are impaired in recognizing a target due to the presence of neighboring objects in the peripheral visual field (Spinelli et al., 2002). Again another hypothesis has been put forward that attributes the problems of dyslexics to a deficit in the perceptual experience of rhythmic timing (Goswami et al., 2002). All these hypotheses about the underlying deficit in dyslexia have mainly been investigated in one research group. On the one hand, this proliferation of causal theories is an exciting and positive feature of contemporary dyslexia research, but on the other hand there is also a dire need for critical replication studies. One of the most recent causal hypotheses of dyslexia attributes the specific problems of dyslexics to a general problem with learning serial order information, or at least to an additional serial learning problem. As learning to read words can be understood as the acquisition of grapheme and phoneme sequences, these researchers suggest that people with dyslexia have a specific deficit in serial order learning. This idea has been investigated by two groups of researchers. First, there is a group of researchers who consider that dyslexics experience difficulties with the consolidation or transfer of serial order information, initially stored in short-term memory (STM), into a stable long-term memory trace. Szmalec et al. (2011) reported empirical evidence for this hypothesis by showing a deficient Hebb repetition effect in dyslexic individuals, even for non-verbal modalities. However, these results could not be confirmed in a recent replication of this study including some methodological improvements (Staels and Van den Broeck, in press). As the results of this replication study show that dyslexics do not suffer from a specific deficit in the consolidation of serial order information in long-term memory, we may wonder whether the problem with storing serial order information is actually not situated in long-term memory but rather in STM. This idea has already been investigated by Martinez Perez and co-authors (Martinez Perez et al., 2012b, 2013) and will also be the main research question of the current study. We will first discuss recent studies and the underlying theoretical assumptions regarding STM deficits in dyslexia. Afterwards, we will also focus on the relationship between STM and reading development.

Martinez Perez and co-authors (Martinez Perez et al., 2012b, 2013) investigated whether the verbal STM deficits often reported in dyslexia can be explained exclusively by the poor phonological processing abilities that characterize dyslexia or whether dyslexics in fact suffer from an additional deficit at the level of serial order STM. Previously, verbal STM deficits in dyslexia had been mainly investigated using tasks that confounded item and serial order information recall (Kramer et al., 2000; Tijms, 2004). Hence, it was not clear whether the poor performance of dyslexic children on these tasks is due to a specific deficit in item STM, order STM, or both. Some recent STM models (Henson, 1998; Brown et al., 1999; Burgess and Hitch, 1999; Gupta, 2003) suggested that verbal item information is stored via temporary activation of phonological and lexo-semantic representations in the language network. Hence, storage of item information would depend directly on the quality of phonological representations in long-term memory and it would only be logical that this is impaired in dyslexia. On the other hand, storage of serial order information would occur via a language-independent system and should therefore be less sensitive to verbal long-term memory representations. Martinez Perez et al. (2012b) argued that if dyslexics would not only show impairments in item STM but would also show impairments in serial order STM, this deficit could not be explained by exclusively referring to poor phonological processing abilities but would be the result of a specific deficit of serial order STM. To investigate this hypothesis they used the distinction between STM for item information and STM for serial order information. In their first seminal study (Martinez Perez et al., 2012b), they administered two tasks designed to maximize either serial order or item retention abilities in a group of dyslexic children, a chronological age-matched group and a reading-level matched group. To assess item retention capacity a non-word delayed repetition task was constructed. In every trial a non-word was presented auditorily to the participants. Participants had to repeat the non-word after a period of time during which they had to perform a distractor task. To assess serial order retention capacity a serial order reconstruction task was administered. For this task in every trial participants had to remember sequences of two to seven real words that were also presented auditorily. Afterwards they were instructed to arrange pictures of these real words in the exact same order as they were presented. The researchers observed that children with dyslexia showed not only impairments on STM for item information but also on STM for serial order information. They concluded that the impairment on STM for serial order information was the most severe since the dyslexic group showed significantly lower performance on the serial order STM task relative to both the age-matched and the reading-level matched control groups, whereas the item STM impairment was only apparent relative to the chronological age-matched control group. In a second study, Martinez Perez et al. (2013) conducted a similar study as the one described previously, but this time they selected a group of adult dyslexics and a chronological age-matched control group without any reading problems. After observing item and serial order STM deficits in the dyslexic group in their first experiment by using the same tasks as in their first study, in a second and third experiment they assessed item and serial order STM retention capacities within the same STM task trying to make a more direct comparison. Additionally, in the third experiment they attempted to equate task sensitivity (difficulty) of item and serial order memory assessments. Again, the authors reported item and serial order STM deficits in the dyslexic group and most importantly they observed that the deficit was stronger at the level of order retention capacities.

As Martinez Perez and co-authors suggest that dyslexia is characterized by a specific deficit in serial order STM, they argue that this impairment in serial order retention capacity could have a negative effect on reading acquisition because learning to read new words can be understood as the acquisition of grapheme and phoneme sequences (Martinez Perez et al., 2012a). In a number of recent studies these researchers have explored the relation among item STM, order STM and language development. They observed that serial order STM capacity is a critical determinant of (oral) vocabulary knowledge and acquisition relative to item STM (Majerus et al., 2006a,b, 2008a,b; Leclercq and Majerus, 2010). Therefore, they argue that serial order STM capacity not only depends on a language-independent system but also appears to be important for the acquisition of new phonological representations (Martinez Perez et al., 2012a). In a recent longitudinal study, Martinez Perez et al. (2012a) investigated if this idea could also be extended to the acquisition of reading and, more precisely, to the acquisition of decoding processes. They investigated the relationship between item STM, order STM and reading development by administering an item and a serial order STM task at the age of kindergarten, and reading decoding ability was assessed 1 year later using a non-word reading task. They reported that serial order STM but not item STM predicted independent variance in reading decoding abilities. Based on the results of this study, the authors argue for a causal role of order STM capacity in reading acquisition.

The current study consists of two experiments. The first experiment will investigate STM deficits in dyslexic children by conceptually replicating the study of Martinez Perez et al. (2012b). However, the method they used will be modified as we believe that some adjustments can improve our study. The second goal of this study is to investigate the relationship between STM and reading acquisition. As STM for order information seems to play a specific role in reading decoding acquisition, order STM capacity could also be important for the acquisition of new long-term orthographic representations as Martinez Perez et al. (2012b) suggest. For that reason, in our second experiment we will use Share's (1995, 1999) self-teaching paradigm to assess reading acquisition. More information about the purpose and the theoretical background of the second experiment will be given in the introduction of Experiment 2. We will first continue by discussing our concerns about a number of methodological issues we encountered in recent studies. Afterwards, we present the methodological improvements we will introduce in our study to address these issues.

First of all we are concerned about the use of a reading-level match (RLM) design. Although this design is still used in some recent studies, it was formally proven that this method often entails methodological problems as it typically confounds diagnostic status with age (cf. Van den Broeck et al., 2010; Van den Broeck and Geudens, 2012; but see Zhou et al., 2014, for a notable exception in which a retrospective RLM-design is used comparing groups when they are at the same age).

In the RLM design of the Martinez Perez study, individuals with reading disabilities were matched with younger typical readers on a measure of reading ability (a text reading test). After this match both groups were compared on the two STM tasks and the researchers concluded that the dyslexic group had a specific deficit for serial order STM. However, Van den Broeck and Geudens (2012) have shown that a RLM design is likely to create processing deficit findings that may in fact be the result of the age differences between groups. One plausible scenario is that the group of older dyslexic readers reached the same reading score in the text reading test as the younger typical readers because they could rely on better word specific knowledge simply because they are older (for evidence see Van den Broeck et al., 2010). The younger normal readers on the other hand probably depended more on their decoding ability in order to reach the same performance level as the older dyslexic readers on the text reading task. This reasoning implies that the RLM matching procedure created an imbalance in decoding ability between both groups. As decoding ability is plausibly associated with the ability to remember order information, it is possible that the younger control group of normal readers only performed better on the serial order STM task as a result of the created imbalance by the design. To be sure that impaired serial-order learning in STM is a genuine characteristic of reading disability, a more direct comparison between typical and disabled readers of the same age is required.

Another methodological problem that occurs in many studies is the fact that researchers rely on the presence of a statistical interaction as evidence for a group related difference. In both studies of Martinez Perez et al. (2012b, 2013), they interpret their results in terms of an interaction effect between task (item memory vs. serial order memory) and group (dyslexic vs. age control group or RL control group). Although this interaction effect was not tested statistically they took the fact that the dyslexic group only showed a significantly lower performance than the reading-level matched control group on the serial order but not on the item STM task, as an indication that the serial order STM deficit was the most severe. The problem with this kind of interpretation is that researchers are usually unaware of the precise form of the relationship between the observed measures and the underlying constructs (Dunn and James, 2003). Therefore, it has been argued that relying on the presence of a statistical interaction as evidence for a qualitative group-related difference is not without problems (Loftus, 1985; Loftus et al., 1987, 2004). Even non-ordinal interaction effects can be made to disappear or reverse by applying a suitable monotonic non-linear transformation to the dependent variable (Bogartz, 1976; Loftus, 1978). This scale-dependency problem is still exacerbated in research where non-experimental variables such as age or pathology are involved because in such situations it is likely that an unspecific general factor influences performance (Kliegl et al., 1994). In the study of Martinez Perez et al. (2012b) one can easily imagine that an overall STM deficit could influence both STM tasks in an unequal manner (for an example of the effects of a general factor, see Van den Broeck and Geudens, 2012, p. 425). As a consequence, an observed interaction effect would be fictitious. This scale-dependency problem also arises when floor or ceiling effects occur in the data (Loftus, 1985).

A last methodological concern in the studies of Martinez Perez et al. (2012b, 2013) is the fact that they did not match their dyslexic and control groups on attentional functioning. Although the authors mention attentional functioning as a potential confounding factor, they refute this possibility by arguing that the order STM task was attentionally not more demanding than the item STM task because error rates were larger in the item STM task than in the order STM task. Furthermore, they convey that dyslexic participants with associated attentional impairment were excluded from the study and therefore they find it unlikely that attentional difficulties could explain the serial order STM impairment in the dyslexic group. However, as the comorbidity of developmental dyslexia and attention deficit disorders (ADHD) is a well-known fact (Araujo, 2012; Boada et al., 2012), and the serial order STM task is very demanding on sustained and focused attention, a serial order STM effect is not necessarily the result of a deficit in serial order retention, but may be attributed to the differential impact of comorbid attention problems on the two memory tasks (see also Wimmer's critique on the automatization deficit hypothesis, Wimmer et al., 1999). For this reason, any research aiming to compare a dyslexic group with a control group on cognitive processing should always make sure that both groups are matched on, or at least controlled for, attentional functioning.

As a result of these three major concerns we will adjust the method used by Martinez Perez et al. (2012b, 2013). To investigate whether dyslexics do suffer from a specific serial order learning deficit in STM it is crucial to make a direct comparison of serial order STM retention capacity when item STM retention capacity is equated between the dyslexic group and a control group of the same age. Indeed, a specific problem in serial order retention can only be proven by directly comparing dyslexic and typical individuals who score equally on the item retention task. When there is considerable overlap between the item retention scores of both groups, state trace analysis (STA) used as an equivalence method is an excellent technique to perform this comparison (see Van den Broeck and Geudens, 2012). In general, STA as a matching technique could be effectively adopted whenever a group showing a particular disorder has to be matched with a typical group, in order to test for a hypothesized specific deficit.

## Materials and methods

### Experiment 1

In Experiment 1 we investigated item and serial order STM capacities in a group of dyslexic and a group of control children matched on IQ and age.

#### Method

***Participants***. A total of 97 children of fourth and fifth grade participated in this study. Thirty-six children had an official diagnosis of dyslexia (20 boys and 16 girls) and 61 were IQ-matched control children without any reading problems (29 boys and 32 girls). Dyslexic participants were either diagnosed by an individual speech therapist or by a specialized center. The diagnoses were all based on three criteria which are used by the Stichting Dyslexie Nederland (2008) (Foundation Dyslexia Netherlands): (1) reading and/or spelling abilities are significantly below the level of performance expected for their age, that is below percentile 10; (2) resistance to instruction despite effective teaching; (3) impairment cannot be explained by extraneous factors, such as sensory deficits. For further validation two norm-referenced Dutch word reading tests that are diagnostic for dyslexia were administered. The first test is the One Minute Test (OMT; Brus and Voeten, 1973), a word reading test in which participants are instructed to read aloud as many words correctly as possible within 1 min. The test consists of 116 real words (nouns, verbs, adjectives, etc.). These words are ordered from lower to higher reading difficulty degree. The second test is the Klepel (Van den Bos et al., 1994), a non-word reading test in which participants are instructed to read aloud as many non-words correctly as possible within 2 min. This test consists of 116 non-words of increasing difficulty. For both reading tests the raw score is the number of words read correctly. In addition to the reading tests, we administered several phonological processing tasks to characterize reading-related skills of both groups. These tests consisted of a phonological awareness task, a phonemic discrimination task and a rapid automatized color and digit naming task (Van den Bos and lutje Spelberg, 2007).

To match the dyslexic and the control groups on IQ, a short-form IQ measure was used including a verbal comprehension subtest (Vocabulary) and a perceptual reasoning subtest (Block design) of the Wechsler Intelligence Scale for Children III (Dutch version) (Wechsler et al., 2005). We also included the Dutch ADHD questionnaire (AVL) (Scholte and Van der Ploeg, 2005) to examine attentional functioning. The questionnaire results in two partial scores: a measure of attentional functioning and a measure of impulsiveness and hyperactivity. As we were only interested in attentional functioning, we only used the partial score on attentional functioning. The questionnaire was completed by the teacher of the participant. Table 1 shows that the experimental group and the control group only differed on the two measures that are diagnostic for dyslexia and on two of the phonological processing tasks. The dyslexic group also showed higher scores on the attentional functioning questionnaire but this difference just missed statistical significance.

**Table 1:** **Characteristics of the dyslexic and control groups (means and standard deviations)**.

	**Controls (*N* = 61)**	**Dyslexics (*N* = 36)**	**Group difference**
Age (years)	10.53 (0.75)	10.75 (0.78)	*p* = 0.192
Word reading test (OMT) (raw score)	**59.44 (9.26)**	**46.06 (10.22)**	*p* = 0.000
Non-word reading test (Klepel) (raw score)	**66.33 (12.44)**	**43.78 (12.29)**	*p* = 0.000
WISC-III block design (standard score)	8.89 (3.15)	8.75 (3.02)	*p* = 0.836
WISC-III vocabulary (standard score)	8.52 (3.16)	8.11 (2.97)	*p* = 0.526
AVL teacher (ADHD questionnaire) (raw score)	6.36 (6.50)	9.28 (7.18)	*p* = 0.054
Phonemic discrimination (raw score)	**95.68 (3.47)**	**91.08 (5.73)**	*p* = 0.000
Phonological awareness (raw score)	**19.37 (3.55)**	**16.31 (4.80)**	*p* = 0.002
Rapid automatized color naming (raw score)	42.11 (9.96)	46.07 (11.00)	*p* = 0.072
Rapid automatized digit naming (raw score)	25.27 (4.12)	26.45 (5.14)	*p* = 0.218

*Values in bold are statistically significant at p = 0.05*.

All children attended regular elementary schools, located in Flanders (Dutch-speaking part of Belgium). Most children were from indigenous families (60%) and children from foreign origins were mainly of Moroccan descent. All children were checked and had sufficient command of the Dutch language to be able to study the Dutch curriculum. Two test assistants were instructed to perform this study.

***Experimental design and procedure***. Testing took place on an individual basis in a quiet classroom at the participant's school. The experimental procedure consisted of two test phases. Each test phase lasted approximately 40 min. All tasks were administered in a fixed order to ensure that the test situation was the same for every participant. During the first session the Block design subtest of the WISC, the OMT, the Klepel, the Serial order STM task and the phonological awareness task were administered. During the second session the Vocabulary subtest of the WISC, the item information STM task, the phonemic discrimination task and the rapid automatized color and digit tasks were administered. All computerized experiments were programmed and presented on a laptop computer using Microsoft Office PowerPoint (2007).

#### Materials

***Phonological processing tasks***.

*Phonemic discrimination task*. Phoneme discrimination abilities were measured using a minimal pair discrimination task. One hundred pairs of nonsense CCV or CCCV syllables were constructed. Fifty pairs of syllables were identical (e.g., sta-sta), 25 pairs differed in one phonetic feature (e.g., dra-pra) and 25 pairs contained a phoneme transposition (e.g., spo-pso). Stimuli were digitally recorded by a female speaker and presented auditorily through headphones. Immediately after presenting a syllable pair, participants were asked to indicate whether both nonsense syllables were identical. The score was the total number of correct answers. Unidimensionality was tested by fitting a one-factor model on categorical data with MPlus 7.11 (Muthén and Muthén, 1998-2012). This model fitted the data well (chi square = 3809.11, *df* = 3827, *p* = 0.585; CFI = 1.00, RMSEA = 0.000). Cronbach's alpha was 0.795, indicating good reliability of the test scores.

*Phonological awareness task*. Phonological awareness abilities were assessed using a position analysis task. For this task, a list of 24 non-words was constructed as stimuli. Every non-word consisted of two syllables and had a length of six or seven letters. Stimuli were digitally recorded by a female speaker and presented auditorily through headphones. Immediately after presenting a non-word participants were asked to repeat the sound that came immediately before or after a target phoneme in the non-word indicated by the experimenter. Half of the items involved identifying the sound before and half after a target phoneme (e.g., which sound comes before “r” in “pristak”?; which sounds comes after “f” in “dreflo”?). The score was the total number of correct answers. A one-factor model fitted the data (chi square = 278.94, *df* = 252, *p* = 0.117; CFI = 0.943, RMSEA = 0.034). Cronbach's alpha was 0.837, indicating good reliability of the test scores.

*Rapid automatized naming*. To assess the speed of lexical access, we used two tasks from the CB and WL test (Van den Bos and lutje Spelberg, 2007), automatic color naming and automatic digit naming. The color naming task involved five colors (black, yellow, red, green, and blue), each presented 10 times. The digit naming task involved five digits (2, 4, 8, 5, 9), each presented 10 times. Each test card contained 50 items of the five colors/digits in random order presented in five columns. In both tasks participants were asked to name the colors/digits as quickly as possible. The score was the time participants needed to name all colors/digits irrespective of response accuracy. Reliability estimates offered by the authors of the test are very good (split half reliability for colors is 0.88 for 4the grade and 0.93 for 5th grade; for digits 0.80 for 4the grade and 0.89 for 5th grade).

***Short-term memory tasks***.

*Item short-term memory task*. As a measure of STM for item information we used a similar task as the delayed item repetition task of Martinez Perez et al. (2012b) and Leclercq and Majerus (2010). A list of 30 CVC non-words was constructed as stimuli (see Appendix A). To maximize the phonological processing demands of this task, stimuli were new and diphone frequency and phonological neighborhood were significantly lower relative to a representative sample of word stimuli. Stimuli were digitally recorded by a female speaker and presented auditorily through headphones to the participant. Each non-word was presented separately. Immediately after the presentation of an item, participants were asked to repeat the non-word to confirm that they had correctly perceived the item. After repeating the item, participants had to count in steps of 2 during 6 s. Afterwards participants were asked to repeat the item again. No feedback was given to the participants. The score was the number of correctly repeated items. A one-factor model fitted the data (chi square = 422.18, *df* = 405, *p* = 0.268; CFI = 0.931, RMSEA = 0.021). Cronbach's alpha was 0.783, indicating good reliability of the test scores.

The task was presented to the child as a game (Leclercq and Majerus, 2010):

You are on an adventure in a castle. The castle has many doors which you have to open. In order to do so, you have to remember a password. You will hear the password through the headphones. The password is a word from a magic language you don't know. Pay close attention to the word and repeat the word out loud. Immediately afterwards start to count out loud by steps of two (0, 2, 4, 6, 8,…) until I say stop and ask you to repeat the password again. Okay?

*Serial order short-term memory task*. As a measure of STM for serial order information we used a similar serial order reconstruction task as Martinez Perez et al. (2012b) and Leclercq and Majerus (2010). Seven names of highly familiar animals (kat, hond, vis, beer, aap, leeuw, kip [cat, dog, fish, bear, monkey, lion, chicken]) were chosen to form lists with lengths ranging from two to seven items. All items were monosyllabic words and every item could only appear once in one trial. The trials were presented by increasing list length, with four trials for each length. The trials were digitally recorded by a female speaker and presented auditorily through headphones to the participant. At the end of each trial, participants received cards of the mentioned animals in random order and were asked to rearrange them in the same order as they were presented. In this task, retention requirements for serial order information were maximized by offering the participant only the cards which contained the pictures that represented the animals that were named in that trial and retention requirements for item information were minimized by using stimuli that were highly frequent and well known in advance. All participants completed all trials and sequence lengths. Since items within a series are correlated, a one-factor model with correlated errors for items belonging to the same series was fitted to the data (chi square = 796.89, *df* = 700, *p* = 0.006; CFI = 0.956, RMSEA = 0.038). Cronbach's alpha was 0.874. However, with correlated errors this index may underestimate or overestimate reliability (Raykov, 1998, 2001). A more conservative estimate is given by the Spearman-Brown coefficient, which was 0.702, indicating at least reasonable reliability of the test scores.

The experimenter presented the task as follows (Leclercq and Majerus, 2010):

Every year, animals from all over the world gather to have a huge race. This year, seven animals are participating: a cat, a dog, a chicken, a lion, a fish, a bear, and a monkey [the experimenter shows the cards of the corresponding animals]. Several races take place. Sometimes only two animals are participating. Sometimes there are three, four, or five animals. At other times, there are big races with six or seven animals. Through the headphones, you will hear someone announce the animal's order of arrival at the finish line, from the first to the last animal. Immediately after I give you the cards with the animals, you have to put the pictures of the animals on the podium in their order of arrival. The animal arriving first has to be put on the highest step and the last one on the lowest step. Okay?

#### Results

**Figure 1:**
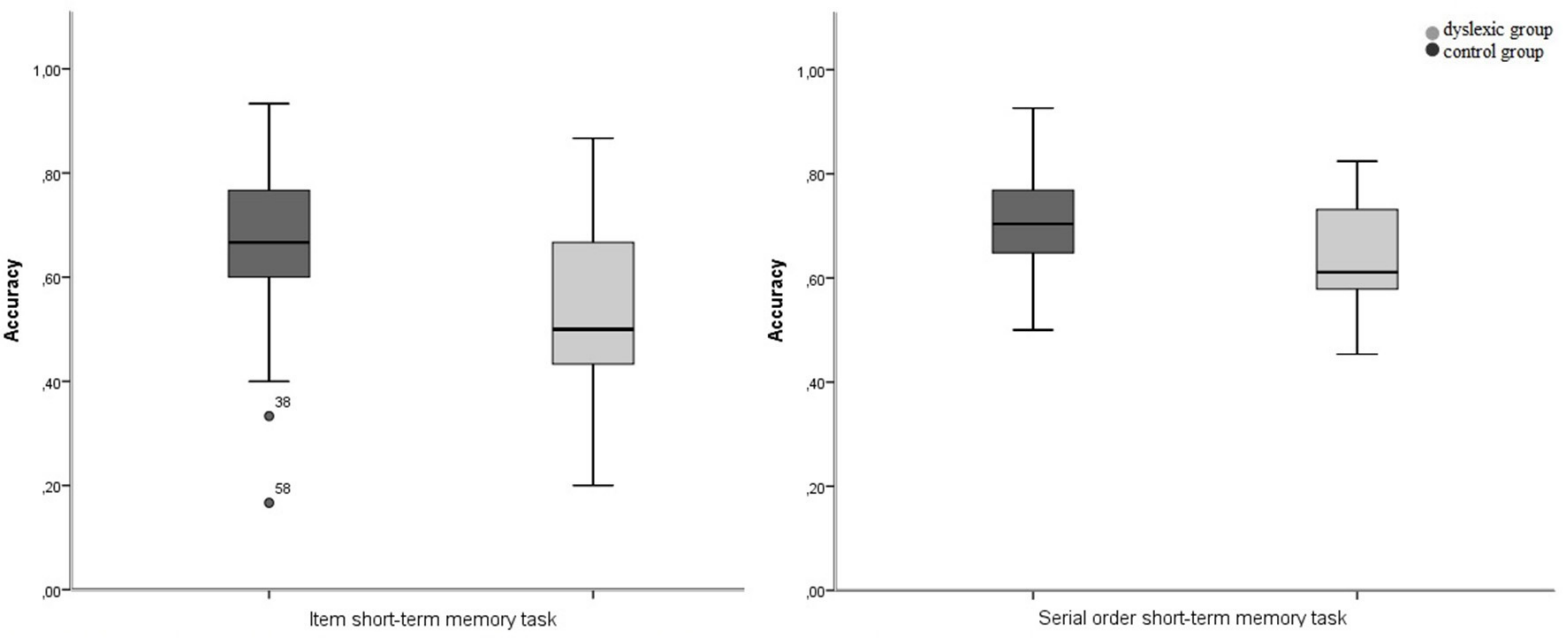
**Boxplot of performance on the item and serial order short-term memory tasks as a function of group (proportion correct)**.

**Figure 2:**
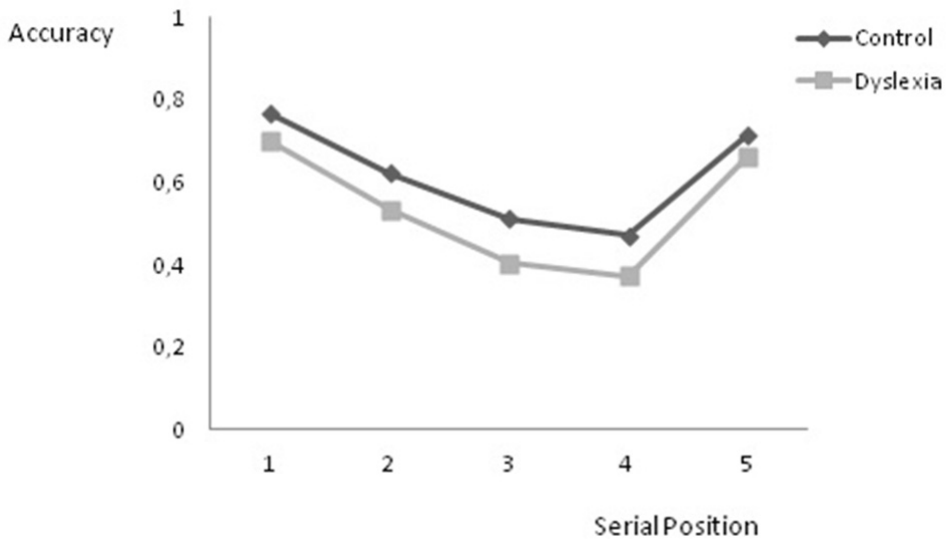
**Proportion of correct responses on the serial order short-term memory task as a function of group and serial position**.

First we analyze our data exactly as Martinez Perez et al. (2012b) did in their study. Afterwards we will address the methodological issues we mentioned before. For the item STM task we determined the proportion of items correctly repeated as the dependent variable (Figure 1). The mean proportion of items correctly repeated was significantly higher in the control group (67%) than in the dyslexic group (53%), *t*_(95)_ = 4.192, *p* = 0.000. For the serial order STM task we determined the proportion of correctly placed items by pooling over all trials as the dependent variable (Figure 2). The mean proportion of items correctly placed over all trials was significantly higher in the control group (71%) than in the dyslexic group (64%), *t*_(95)_ = 3.200, *p* = 0.002.

We also analyzed performance on the serial order STM task as a function of serial position to obtain a qualitative view of the serial order retention process. As we noticed that all participants obtained a maximum score on all trials of with length of 2, 3, and 4, we restricted our analyses to list lengths of 5–7 to avoid floor effects. To increase the sensitivity of the analysis we combined serial positions 4 and 5 of list length 6 and serial positions 3 and 4 as well as 5 and 6 of list length 7. This means that we used the scores on the five positions of list length 5, and for list length 6 five scores were assembled (score on items in position 1, score on items in position 2, score on items in position 3, mean score on items in positions 4 and 5 and score on items in position 6) and for list length 7 also five scores were constructed (total score on items in position 1, total score on items in position 2, mean score on items in positions 3 and 4, mean score on items in positions 5 and 6 and score on items in position 7). Consequently, scores on five serial positions were entered into the analysis. Figure 2 shows the proportion of correct responses as a function of group and serial position. A repeated measurements ANOVA revealed significant main effects of group, *F*_(1, 95)_ = 8.610, *p* = 0.004, and serial position, *F*_(4, 92)_ = 176.532, *p* = 0.000. No group by serial position interaction effect was found, *F*_(4, 92)_ = 1.372, *p* = 0.250.

In order to verify whether reading disability affected one STM task after statistically controlling for the other memory task, we conducted analyses of covariance (ANCOVA). By entering a covariate into an ANOVA the covariance of this variable with the other independent variable(s) is removed before the influence on the dependent variable is determined. For the item STM task the effect of group remained significant when the performance on the serial order STM task was entered as a covariate, *F*_(1, 94)_ = 8.587, *p* = 0.004. This means that even if the reading groups are statistically equated on the performance on the serial order STM task, the effect of group on the item STM task still remains significant. This result was in line with the results of Martinez Perez et al. (2012b). However, in contrast with their results, for the serial order STM task the effect of group disappeared when the performance on the item STM task was entered as a covariate, *F*_(1, 94)_ = 1.906, *p* = 0.171. This implies that the difference between the dyslexic and control group on the serial order STM task is no longer statistically significant when differences on the item STM task are taken into account. This result demonstrates that the item STM task and the serial order STM task do not measure entirely independent processes.

Martinez Perez et al. (2012b) also predicted that item STM but not order STM should be related to phonological processing measures. In order to investigate their prediction, we performed a set of correlation analyses. We only report the correlations observed in the total group (virtually the same results were observed for the dyslexic and control groups when analyzed separately). The results of these analyses not only reveal significant correlations between item STM and both phonological tasks (phonemic discrimination and phonological awareness), but also between serial order STM and the phonological tasks (see Table 2). In fact, the latter were even somewhat larger. No significant correlations were observed between item STM or serial order STM and rapid automatized naming tasks. In contrast to the results of Martinez Perez et al. (2012b), our data show clearly that both item STM and serial order STM are related to phonological processing measures. Remarkably, serial order STM was significantly related to both IQ-subtests, especially with block design, whereas item STM was not.

**Table 2:** **Correlations and partial correlations controlling for age (between brackets) between short-term memory tasks, phonological processing tasks, reading tests and IQ-subtests for all participants (*N* = 97)**.

	**Item short-term memory**		**Serial order short-term memory**	
Phonemic discrimination	**0.390 (0.397)**	*p* = 0.000	**0.443 (0.461)**	*p* = 0.000
Phonological awareness	**0.466 (0.474)**	*p* = 0.000	**0.546 (0.561)**	*p* = 0.000
Rapid automatized color naming	−0.061 (−0.042)	*p* = 0.553	−0.161 (-0.087)	*p* = 0.116
Rapid automatized digit naming	−0.115 (−0.116)	*p* = 0.262	−0.094 (-0.113)	*p* = 0.358
Word reading test (OMT) (raw score)	**0.473 (0.476)**	*p* = 0.000	**0.400 (0.426)**	*p* = 0.000
Non-word reading test (Klepel) (raw score)	**0.448 (0.461)**	*p* = 0.000	**0.359 (0.387)**	*p* = 0.000
WISC-III block design (standard score)	0.063 (0.061)	*p* = 0.540	**0.387 (0.357)**	*p* = 0.000
WISC-III vocabulary (standard score)	0.150 (0.165)	*p* = 0.144	**0.236 (0.228)**	*p* = 0.020

*Values in bold are statistically significant at *p* = 0.05*.

#### State trace analysis

We now analyze our data using STA as an improved matching design. In the analysis we present here, another methodological improvement is introduced. The dyslexic group and the control group were not only matched on intellectual functioning but on attentional functioning as well. By discarding 20 control subjects and no dyslexic subject from the initial sample, we obtained similar distributions for both groups on the attention questionnaire. As Table 3 indicates, after this additional matching, the newly formed groups of dyslexic children and control children only differed on the two measures that are diagnostic for dyslexia and on the two measures of phonological processing.

**Table 3:** **Characteristics of the dyslexic and control groups after matching on attentional functioning (means and standard deviations)**.

	**Controls (*N* = 41)**	**Dyslexics (*N* = 36)**	**Group difference**
Age (years)	10.63 (0.76)	10.75 (0.78)	*p* = 0.530
Word reading test (OMT) (raw score)	**59.34 (9.82)**	**46.06 (10.22)**	*p* = 0.000
Non-word reading test (Klepel) (raw score)	**66.32 (12.51)**	**43.78 (12.29)**	*p* = 0.000
WISC-III block design (standard score)	8.68 (3.09)	8.75 (3.02)	*p* = 0.924
WISC-III vocabulary (standard score)	8.37 (2.95)	8.11 (2.97)	*p* = 0.707
AVL teacher (ADHD questionnaire)	9.22 (6.14)	9.28 (7.18)	*p* = 0.970
Phonemic discrimination	**95.35 (2.65)**	**91.08 (5.73)**	*p* = 0.000
Phonological awareness	**18.83 (3.85)**	**16.31 (4.80)**	***p* = 0.013**
Rapid automatized color naming	42.91 (11.26)	46.07 (11.00)	*p* = 0.217
Rapid automatized digit naming	25.34 (4.62)	26.45 (5.14)	*p* = 0.325

*Values in bold are statistically significant at *p* = 0.05*.

Using STA, serial order STM performance can be compared directly between the two groups at each level of item STM performance. Compared to the traditional method of interpreting interaction effects by comparing group differences across tasks, STA is more sensitive to detect a specific serial order STM deficit because by matching dyslexic and control subjects on item STM performance, both groups are equated on STM processing without involving the crucial serial order information. After inspecting that both groups show substantial overlap on the item STM performance, serial order STM is regressed on performance on item STM separately for the dyslexic group and the control group. It is tested whether a single line is suitable to explain the data (the null model not including reading group) or whether two different lines (one for dyslexic children and one for control children) are needed to describe the relation between serial order STM performance and item STM performance (the full model). If a single line would fit the data, this would imply that the relation between serial order STM performance and item STM performance is not affected by dyslexia. If, on the other hand, two lines would fit our data better, and the one for dyslexic children would be situated lower than the one for control children, this would be direct evidence for a specific serial order STM deficit in dyslexic children^1^.

**Figure 3:**
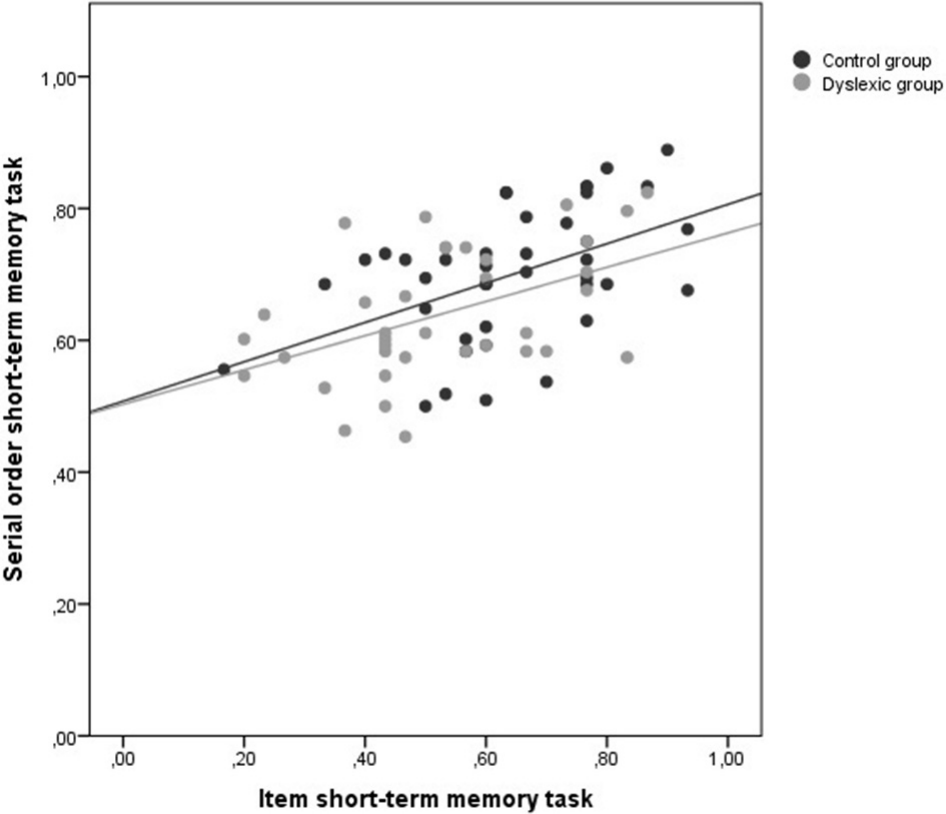
**State trace analysis with performance on the item STM task on the x-axis and performance on the serial order STM task on the y-axis (proportions correct)**.

In this analysis, for each participant item and serial order scores were averaged and then plotted against each other (see Figure 3). Then, we tested in a hierarchical regression analysis whether group contributed significantly to serial order STM after including item STM performance in the regression equation. This analysis showed that adding group as a predictor doesn't significantly improve fit [*R*^2^ null model = 0.271 *R*^2^ full model = 0.285; Δ*R*^2^ = 0.014; *F* change_(1, 74)_ = 1.488; *p* = 0.226].

Hence, the null hypothesis—that the state trace curves for the typical and for the disabled readers do not differ—could not be rejected. This means that if there was no difference between the curves of the two groups (if H0 is true) in reality, the probability of finding a difference as large as or even larger than in our sample is 0.23. As STA entails that the null hypothesis is in fact a substantive hypothesis, this number does not seem really convincing. Note that a non-significant result due to a lack of power must not be confused with support for the null hypothesis. What we really want to know is the probability that the null hypothesis or the alternative hypothesis is true given the observed data (the inverse probability). To this end, a Bayesian analysis was performed in which the probability of the null model was compared to the probability of the full model given the data and given the assumption that no model was preferred above the other. For this comparison Bayesian Information Criteria (BIC) were calculated for both models. The BIC has been proposed by Raftery (1996) as an index to assess the overall fit of a model and allows a comparison of models (see also Long, 1997). Given that BIC assesses whether the model fits the data sufficiently well to justify the number of parameters that are used, the model with the lowest BIC is the best fitting, yet parsimonious model. The BIC-values indicated that the null model fitted the data best (BICnull = −144.88 and BICfull = −142.13). Based on the difference between these BIC-values the “Bayesian factor” could be calculated (Kass and Raftery, 1995). The Bayesian factor is equal to the posterior odds in favor of the most likely hypothesis. The posterior odds of the null model (MN) relative to the full model (MF) equal:

$$\frac{\text{Pr}\left({\text{M}}_{\text{N}}\text{/Observed Data}\right)}{\text{Pr}\left({\text{M}}_{\text{F}}\text{/Observed Data}\right)}$$

The Bayesian factor favoring the null model was 2.75. According to the criteria proposed by Kass and Raftery (1995), the data provided “positive” evidence for the null hypothesis.

### Experiment 2

The results of a longitudinal study of Martinez Perez et al. (2012a) indicate that order STM abilities in kindergarten predict later reading decoding abilities. They make the stimulating suggestion that besides this specific contribution of order STM in reading decoding processes, order STM capacity might also be important for the acquisition of orthographic representations of new written words in long-term memory, and hence could be a major factor in reading development. Therefore, in this second experiment we will focus on the prediction of a differential role of item STM and serial order STM capacity in the orthographic learning of primary school readers over the entire range of reading ability. Additionally, we test the hypothesis that serial order STM would be impaired in a group of relatively poorer readers.

Item STM and order STM abilities were measured using the same tasks as used in Experiment 1. We also administered the same phonological awareness task as used in Experiment 1 to investigate whether item STM and serial order STM are related to phonological processing abilities. To assess reading and spelling ability, two word reading tests and a spelling test were administered. In addition, orthographic learning was assessed using Share's (1999) self-teaching paradigm. According to the self-teaching hypothesis (Share, 1995, 1999, 2004) children are able to acquire orthographic representations independently from an external teacher. Orthographic learning, the process through which orthographic representations are formed, consists of two independent processes. First, an unfamiliar written word is phonologically recoded into its spoken form by using known grapheme-phoneme associations. If this step succeeds, the phonological code of the word will be mapped onto its orthographic counterpart, establishing word-specific knowledge of the spelling of the new word. The self-teaching hypothesis was supported in a number of studies using an experimental paradigm adapted from Reitsma (1983). In these studies, target words were presented several (four or six) times in a natural text (Share, 1999). These targets were pseudowords representing a fictitious place, animal or fruit. Every pseudoword (e.g., yait) had an alternative homophone spelling (e.g., yate) and in each case only one spelling, the target spelling, was presented to the participant. Each participant was asked to read aloud the stories and to answer some questions about the content of the stories afterwards to ensure that they understood the text. Following Reitsma's (1983) procedure, orthographic learning was assessed 3 days after text reading using three types of measures: an orthographic choice task, a naming task and a spelling task. For the first measure, orthographic choice, children were asked to select the correct spelling of the target among the two homophone spelling alternatives. Secondly, children were instructed to read a list of words appearing on a computer screen as quickly and accurately as possible. The list of words contained all targets and their homophone spellings. Finally, the last test of orthographic learning required children to reproduce the target spelling in writing. The general outcome of studies based on this paradigm was that 3 days after independently reading the stories aloud, target spellings were recognized more often, named faster and spelled more accurately than their alternate homophone spellings. Relatively few successful identifications of an unfamiliar word appeared to be sufficient to acquire orthographic representations for young children (Hogaboam and Perfetti, 1978; Reitsma, 1983; Manis, 1985) and also for poor readers Staels and Van den Broeck (2013). Although most evidence for the self-teaching hypothesis is based on oral reading, recent studies have shown the appearance of orthographic learning in silent reading as well (Bowey and Muller, 2005; Bowey and Miller, 2007; de Jong and Share, 2007; de Jong et al., 2009). These findings provide important support for orthographic learning occurring in independent daily reading.

#### Method

***Participants***. One hundred and eighty eight third (38), fourth (93), and fifth (57) grade children participated in this study (96 boys, 92 girls). All children of entire classes were selected to participate in this study. Their age ranged from 7 years 11 months to 11 years 10 months, with a mean age of 9 years 6 months. All children attended regular elementary schools, located in several regions in Flanders and in urban and rural areas. Most children were from indigenous families (73%) and for 83% of the children their home language was Dutch. All children were checked and had sufficient command of the Dutch language to be able to study the Dutch curriculum. Four test assistants were instructed to perform this study.

***Experimental design and procedure***. The experimental procedure consisted of two phases. In the first test session a spelling test, based on the PI-dictee (Geelhoed and Reitsma, 1999) was administered for the entire class group. Afterwards the reading phase of Share's (1999) self-teaching paradigm was carried out. All students in the class were instructed to read all eight stories once in silence. They were encouraged to read the texts very attentively as they were warned that immediately after each text, two questions would be posed about the content of the stories to check text comprehension. All students were given enough time to read the texts and answer the questions at their own pace. This session lasted approximately 30 min. The second test phase took place on an individual basis in a quiet classroom at the participant's school. Two Dutch reading tests (OMT; Brus and Voeten, 1973 and the Klepel; Van den Bos et al., 1994), two measures of orthographic learning (orthographic choice task and orthographic spelling task), two STM tasks (serial order STM task and item STM task), the phonological awareness task and the Vocabulary subtest of the WISC-III (Dutch version) (Wechsler et al., 2005) were administered. All tasks were run in the indicated fixed order, except for the order of the two orthographic learning tasks which was counterbalanced across participants. All computerized experiments were programmed and presented on a laptop computer using Microsoft Office PowerPoint (2007).

We also included the Dutch ADHD questionnaire (AVL) (Scholte and Van der Ploeg, 2005) to examine attentional functioning in our experimental procedure. As we were only interested in attentional functioning, we only used the partial score on attentional functioning of the questionnaire. The questionnaire was completed by the teacher of the participant.

#### Materials

***Spelling task***. A spelling task was constructed based on the Dutch spelling test PI-dictee (Geelhoed and Reitsma, 1999). As children of the third, fourth and fifth grade participated in this study fifteen words were selected varying in difficulty. Five words to assess spelling in every grade were chosen from the PI-dictee. For every word a sentence in which the word occurs was read aloud by the test assistant. The word the participants had to write down was repeated afterwards. The score was the total number of correctly written words. A one-dimensional model fitted the data well (chi square = 97.73, *df* = 90, *p* = 0.271; CFI = 0.993, RMSEA = 0.021). Cronbach's alpha was 0.827, indicating good reliability of the test scores.

***Phonological awareness task***. The same phonological awareness task as in Experiment 1 was used. A one-factor model fitted the data not quite well (chi square = 303.49, *df* = 252, *p* = 0.0145; CFI = 0.929, RMSEA = 0.033). After inspection of the modification indices, a two-factor model was fitted with all items requiring to give the phoneme(s) before the target phoneme loading in one factor, and all items requiring to give the phoneme(s) after the target phoneme loading in another factor (chi square = 256.70, *df* = 251, *p* = 0.389; CFI = 0.992, RMSEA = 0.011). Because the correlation of both factors was quite high (*r* = 0.672) and because both factors showed very similar correlations with all other tests, we decided to treat this test as measuring one concept. Cronbach's alpha was 0.835, indicating good reliability of the test scores.

***Short-term memory tasks***.

*Item short-term memory task*. The same item STM task as in Experiment 1 was used. Although a one-factor model with all items included fitted the data reasonably well, inspection of the factor loadings revealed that one item “pob” did not load significantly in this factor. Probably the reason for this is the fact that this item is phonetically not a non-word in Dutch. After removing this item in a one-factor model a nice fit was obtained (chi square = 380.38, *df* = 377, *p* = 0.442; CFI = 0.995, RMSEA = 0.007). Cronbach's alpha was 0.803, indicating good reliability of the test scores.

*Serial order short-term memory task*. The same serial order STM task as in Experiment 1 was used. Since items within a series are correlated, a one-factor model with correlated errors for items belonging to the same series was fitted to the data (chi square = 802.84, *df* = 700, *p* = 0.004; CFI = 0.955, RMSEA = 0.028). Cronbach's alpha was 0.90. However, with correlated errors this index may underestimate or overestimate reliability (Raykov, 1998, 2001). A more conservative estimate is given by the Spearman-Brown coefficient, which was 0.639, indicating at least reasonable reliability of the test scores.

***Self-teaching phase***. The self-teaching phase of this study is based on Share's (1999) self-teaching paradigm. Eight short Dutch texts, similar to Share's (1999) stories, were composed for this study. All texts were adjusted to the overall reading level of the participants and ranged in length from 65 to 148 words (mean length 94). Targets were eight novel letter strings (pseudowords) representing a fictitious animal or person. Each target included two phonemes that could be represented by two alternate graphemes. These alternate letters occurred at various positions across target strings. The eight designed target quadruplets had a length of one or two syllables and ranged from five to seven letters. Four versions of each story were created, each employing one of the four homophone spellings of the following target quadruplets: Bleip/Blijp/Bleib/Blijb; Traug/Trauch/Troug/Trouch; Drouft/Droufd/Drauft/Draufd; Reilt/Reild/Rijlt/Rijld; Weipsik/Wijpsik/Weipzik/Wijpzik; plijmap/pleimap/plijmab/plijmab; Kauwand/Kouwand/Kauwant/Kouwant; Hichtop/Higtop/Hichtob/Higtob. Each target appeared six times in one of the eight texts and once in one of the two comprehension questions. Texts were presented separately on A4 paper.

***Orthographic learning tasks***. Orthographic learning was assessed 1–7 days after the self-teaching phase with an orthographic choice task and a spelling task (Share, 1999).

*Orthographic choice task*. Participants were first asked a question to recall the target word (e.g., “Do you remember the name of the monkey who wanted to move to the zoo in the story?”). Each participant was then shown the four alternatives of the target word. The examiner presented a sheet of paper to the participant with the four alternate spellings of the target words written next to each other. The words were written in a random order. Participants were asked to choose the spelling of the pseudoword they had read in the story. The score on this task was the total number of items correctly chosen with a maximum score of eight.

*Spelling task*. Participants were asked to spell the target spelling of the animal or person they had read about in the story. If the participant could not recall the name of the target, the name was provided by the examiner. The score was the sum of the number of target graphemes written correctly within all pseudowords. This means that for every target word a score of 0, 1, or 2 was given with a maximum score of 16 on the entire task.

#### Results

**Figure 4:**
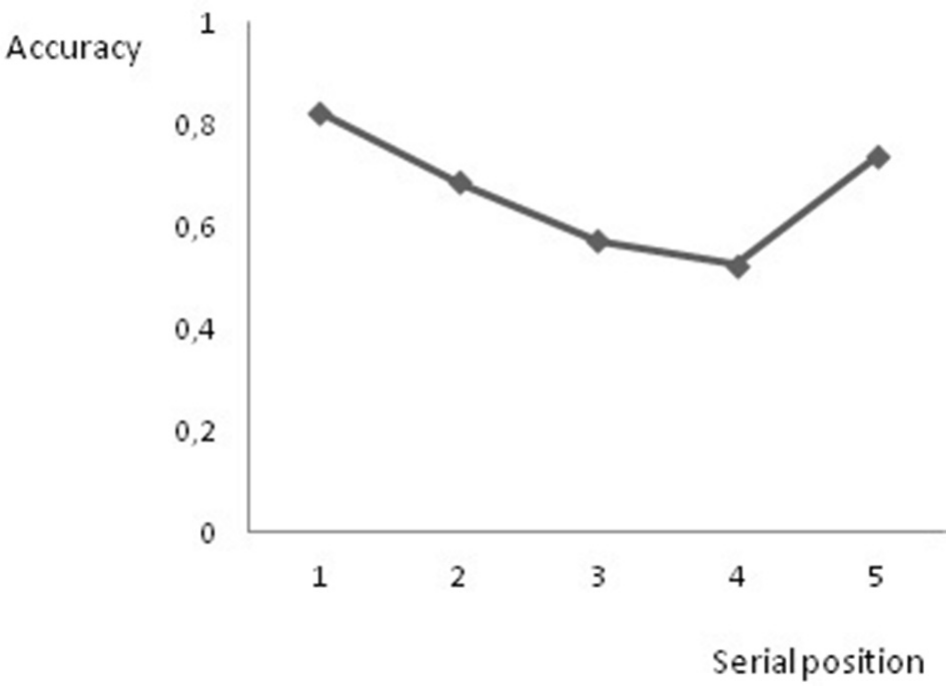
**Proportion of correct responses on the serial order short-term memory task as a function of serial position**.

***Item and serial order STM***. For the item STM task we determined the proportion of items correctly repeated. The mean proportion of items correctly repeated was 72%. For the serial order STM task the proportion of correctly placed items was determined by pooling over all trials. The mean proportion of items correctly placed over all trials was 73%. As in our first experiment, we performed an analysis on performance as a function of serial position to obtain a qualitative view of the serial order retention process. Again, we restricted our analyses to list lengths 5–7 and we combined serial positions 4 and 5 of list length 6 and serial positions 3 and 4 as well as 5 and 6 of list length 7. Consequently, five serial positions were entered into the analysis (see Figure 4).

*Orthographic learning*. Because we used a silent reading procedure, it was not possible to determine the proportion of correctly decoded pseudowords. On the comprehension questions the mean proportion of correct answers was 93%, indicating that these questions were simple, yet effective to check whether the children read the texts carefully.

The dichotomous categorical (success/failure) data from the orthographic choice tasks were tested using a one-tailed *t*-test for the divergence of the predetermined chance-level proportion of 25%. We determined a chance level of 25% for the orthographic choice task because participants were forced to choose one of the four presented homophone foils. A random pick would therefore yield a score of 25%. For the orthographic spelling task participants were asked to write down the target pseudoword. As the pseudoword was provided auditorily by the experimenter, participants had a chance of 50% to write each of the two homophone graphemes that occurred in the pseudoword correctly. Hence, they had a chance of 25% to write both homophone graphemes correctly in every pseudoword. The overall proportion of correct choices on the orthographic choice task was 0.43 (*SD* = 0.20), which was significantly larger than the chance level proportion correct of 0.25, *t*_(186)_ = 12.374, *SE* = 0.015, *p* = 0.000, one-tailed. The proportion of correctly spelled target graphemes in the spelling task was 0.64 (*SD* = 0.15), which was also significantly larger than the proportion correct of 0.25, *t*_(186)_ = 34.704, *SE* = 0.011, *p* = 0.000, one-tailed. Summarized, these results demonstrate that target spellings were recognized more often and correctly spelled more often than chance level.

To investigate the relationship between STM and reading acquisition we performed a number of correlation analyses. Table 4 shows that of the two orthographic learning measures only the orthographic choice task is related to item STM capacity. Serial order STM is clearly not related to orthographic learning. We even found a small but significant negative effect of serial order STM on the orthographic spelling task after controlling for item STM (beta = –0.168, *t* = −2.17, *p* = 0.031). Table 4 also reveals significant correlations between both STM tasks and the phonological awareness task. The correlation between the phonological awareness task and serial order STM is almost as high as the correlation between phonological awareness and item STM.

**Table 4:** **Correlations and partial correlations controlling for age (between brackets) between short-term memory tasks, orthographic learning tasks, spelling and reading tasks, vocabulary knowledge, and phonological awareness task for all participants (*N* = 188)**.

**(Raw scores)**	**Item STM**		**Serial order STM**	
Orthographic choice task	**0.160 (0.153)**	*p* = 0.028	–0.010 (–0.011)	*p* = 0.887
Orthographic spelling task	0.065 (0.055)	*p* = 0.378	–0.129 (–0.132)	*p* = 0.079
Phonological awareness	**0.477 (0.462)**	*p* = 0.000	**0.440 (0.444)**	*p* = 0.000
Spelling task	**0.337 (0.312)**	*p* = 0.000	**0.203 (0.213)**	*p* = 0.005
WISC-III vocabulary	**0.231 (0.198)**	*p* = 0.000	**0.282 (0.304)**	*p* = 0.000
Word reading test (OMT)	**0.434 (0.408)**	*p* = 0.000	**0.206 (0.206)**	*p* = 0.004
Non-word reading test (Klepel)	**0.356 (0.329)**	*p* = 0.000	0.101 (0.095)	*p* = 0.167

*Values in bold are statistically significant at *p* = 0.05*.

***Item and serial order STM in relatively poor readers and typical readers***. We divided the group of participants in two groups based on their reading level. To define these groups, the mean of the standard scores of the two Dutch reading tests (OMT; Brus and Voeten, 1973 and the Klepel; Van den Bos et al., 1994) was taken. Participants who scored one standard deviation below this mean were assigned to the group of poor readers. All other participants were assigned to the group of typical readers. Table 5 shows that the two groups differ on the two reading tests, on the attention questionnaire, on the phonological awareness task, the spelling task and on both STM tasks.

**Table 5:** **Characteristics of the group of poor readers and the group of typical readers (means and standard deviations)**.

	**Typical readers (*n* = 158)**	**Poor readers (*n* = 30)**	**Group difference**
Age (years)	9.44 (0.85)	9.71 (0.98)	*p* = 0.127
Word reading test (OMT) (raw score)	**59.92 (11.73)**	**36.70 (8.85)**	*p* = 0.000
Non-word reading test (Klepel) (raw score)	**62.94 (15.61)**	**32.23 (10.41)**	*p* = 0.000
WISC-III vocabulary (standard score)	10.68 (2.87)	9.90 (2.54)	*p* = 0.168
AVL teacher (ADHD questionnaire) (raw score)	**3.50 (4.73)** (*N* = 156)	**7.00 (5.96)** (*N* = 30)	*p* = 0.000
Phonological awareness	**20.97 (3.38)**	**17.53 (5.08)**	*p* = 0.000
Spelling task	**9.04 (3.26)**	**5.97 (2.68)**	*p* = 0.000
Orthographich choice task	3.50 (1.63)	3.10 (1.32)	*p* = 0.218
Orthographic spelling task	10.32 (2.49)	9.83 (2.30)	*p* = 0.321

*Values in bold are statistically significant at *p* = 0.05*.

The mean proportion of items correctly repeated in the item STM task was significantly higher in the typical readers group (75%) than in the poor readers group (58%), *t*_(186)_ = −5.371, *p* = 0.000. For the serial order STM task, the mean proportion of items correctly placed over all trials was significantly higher in the typical readers group (74%) than in the poor readers group (69%), *t*_(186)_ = −2.541, *p* = 0.012.

In order to verify whether reading ability affected one STM task after statistically controlling for the other memory task, we conducted analyses of covariance (ANCOVA). For the item STM task as the dependent variable, the effect of group remained significant when the performance on the serial order STM task was entered as a covariate, *F*_(1, 185)_ = 22.339, *p* = 0.000. In contrast, for the serial order STM task as the dependent variable, the effect of group disappeared when the performance on the item STM task was entered as a covariate, *F*_(1, 185)_ = 0.728, *p* = 0.395. These results are similar to the results of our first experiment and demonstrate that the item STM task and the serial order STM task do not measure entirely independent processes. More specifically, when differences on the item STM task are taken into account, serial order STM differences between the reading groups disappear.

To directly compare serial order STM performance in both groups for each level of item STM performance, we analyze our data again using STA. First, the poor readers group and the typical readers group were not only matched on intellectual functioning but on attentional functioning as well. By discarding 91 normal readers and no poor readers from the initial sample, we obtained similar distributions for both groups on the attention questionnaire. As Table 6 shows, after this additional matching, the newly formed groups of poor readers and typical readers differed on the two measures that are diagnostic for dyslexia, on the phonological awareness task and on the spelling task.

**Table 6:** **Characteristics of the group of poor readers and the group of typical readers after matching on attentional functioning (means and standard deviations)**.

	**Normal readers (*N* = 67)**	**Poor readers (*N* = 30)**	**Group difference**
Age (years)	9.36 (0.90)	9.71 (0.98)	*p* = 0.085
Word reading test (OMT) (raw score)	**56.64 (10.89)**	**36.70 (8.85)**	*p* = 0.000
Non-word reading test (Klepel) (raw score)	**60.58 (15.29)**	**32.23 (10.41)**	*p* = 0.000
WISC-III vocabulary (standard score)	9.70 (3.12)	9.90 (2.54)	*p* = 0.761
AVL teacher (ADHD questionnaire) (raw score)	7.05 (4.70)	7.00 (5.96)	*p* = 0.968
Phonological awareness	**20.96 (3.90)**	**17.53 (5.08)**	*p* = 0.000
Spelling task	**8.21 (3.21)**	**5.97 (2.68)**	*p* = 0.001
Orthographich choice task	3.18 (1.57)	3.10 (1.32	*p* = 0.821
Orthographic spelling task	9.85 (2.73)	9.83 (2.30)	*p* = 968

*Values in bold are statistically significant at *p* = 0.05*.

**Figure 5:**
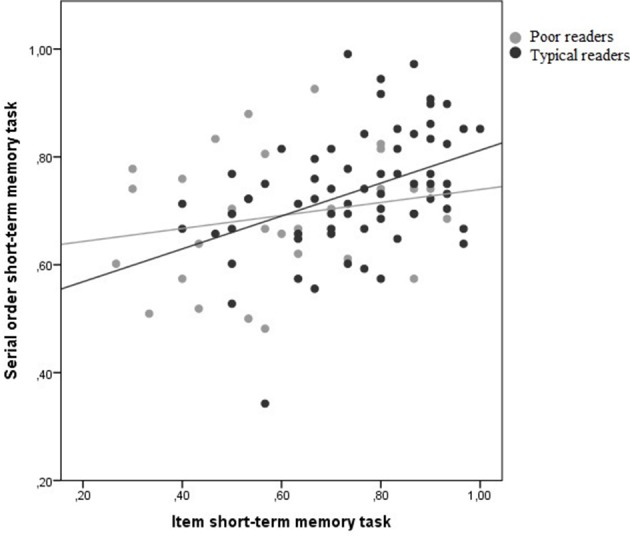
**State trace analysis with performance on the item STM task on the x-axis and performance on the serial order STM task on the y-axis (proportions correct)**.

After inspecting that both groups show substantial overlap on the item STM performance, serial order STM is regressed on performance on item STM separately for the poor reading group and the typical reading group. In this analysis, for each participant item and serial order scores were averaged and then plotted against each other (see Figure 5). Then, we tested in a hierarchical regression analysis whether group contributed significantly to serial order STM after including item STM performance in the regression equation. This analysis showed that adding group as a predictor doesn't significantly improve fit [*R*^2^ null model = 0.146 *R*^2^ full model = 0.147; Δ*R*^2^ = 0.001; *F* change_(1, 94)_ = 0.104; *p* = 0.747]. Hence, the null hypothesis—that the state trace curves for the typical and for the disabled readers do not differ—could not be rejected. A Bayesian analysis was performed in which the probability of the null model was compared to the probability of the full model given the data and given the assumption that no model was preferred above the other. The BIC-values indicated that the null model fitted the data best (BICnull = 759.57 and BICfull = 764.04). Based on the difference between these BIC-values the “Bayesian factor” could be calculated (Kass and Raftery, 1995). The Bayesian factor favoring the null model was 4.47. According to the criteria proposed by Kass and Raftery (1995), the data provided “positive” evidence for the null hypothesis.

## Discussion

The main aim of Experiment 1 was to try to replicate the specific serial order STM deficit in dyslexic readers as reported by Martinez Perez and co-authors (Martinez Perez et al., 2012b, 2013). As our results showed, we were unable to detect a specific deficit in serial order STM capacity in dyslexic children. However, a potential limitation of this experiment is that many participants were in fact bilingual. Although all children were proficient in Dutch, as they typically attended Flemish (Dutch speaking) schools from first grade on, it is possible that the reading problems of the bilingual dyslexic children were partly affected by their bilingual status. To examine this potential confound of language background, the interaction effect of diagnostic category with language spoken at home (coded 1 for Dutch speaking children and 0 for all other children) was tested. This effect was not significant (*p* = 0.33) implying that the effect of being dyslexic or not on serial order STM performance was not different for bilingual children and monolingual Dutch speaking children. Moreover, when only the Dutch speaking children were included in the hierarchical regression analysis (13 dyslexic and 13 control), the group factor (being dyslexic or not) did not contribute significantly after controlling for item STM (*p* = 0.14). Likewise, in Experiment 2 no specific serial order STM deficit was detected in poor readers. Given that both experiments were designed with more power (larger samples sizes) than those of Martinez Perez et al. and with a direct comparison between item and serial order STM performance, using STA, we can be confident to conclude that the impairments in STM often reported in dyslexia are not due to a specific impairment in the retention of serial order information. Although it would be difficult to speculate on why the results of Martinez Perez and co-authors (Martinez Perez et al., 2012b, 2013) did show such a dyslexic deficit and our experiments did not, it seems that the use of STA enabled a more direct comparison of serial order STM performance after equating item STM performance between groups. The lack in their studies of a more stringent match on attentional functioning does not seem to be an important factor as the conclusions of our own studies were not different when we analyzed the entire original group of participants without matching on attention (not reported). Probably the match on item STM performance which is inherent in STA is already sufficient to match the groups on attentional functioning. The apparent instability of a serial order STM deficit in dyslexic individuals is also evident from two recent studies. Hachmann et al. (2014) found evidence for such a deficit in dyslexic adults whereas Binamé and Poncelet (2014) reported an item STM deficit in adult poor spellers as well as a serial order STM deficit. Based on the data of these authors we found that the serial order STM deficit disappeared after controlling for item STM performances^2^. Moreover, these authors could not find a deficient Hebb repetition effect in their sample of poor spellers. Everything being taken into account, would a serial order STM deficit in dyslexics be a robust phenomenon, one would have expected a more consistent pattern of results.

A second important conclusion that follows from both reported experiments is that the measurement of serial order STM is at least as strongly related to phonological processing as is the measurement of item STM. What do these results tell us about theories assuming the separability of both STM components, and about the role of phonology as a basis of reading (dis)ability? First, when the serial order STM task bears a phonological component (names of animals), even if the phonological demands are minimized, the relationship with phonological abilities proves to be at least as strong as is the case for item STM. This implies that the assumed disconnection between item and serial order STM processes does not coincide with the phonology/non-phonology distinction. On the other hand, our results are in agreement with the idea of Martinez-Perez and co-authors that both STM processes are partly independent. As the correlations between item STM performance and serial order STM performance are far below the reliability estimates of both tasks (*r* = 0.50 in Experiment 1 and *r* = 0.34 in Experiment 2), it is clear that serial order STM scores contain some unique variance that is not accounted for by item STM scores. There is both behavioral and neurological evidence that item information and sequence information are coded distinctly in STM. The recall of verbal item information is shown to be affected by psycholinguistic properties such as word frequency and semantic content, while recall of the order of the items is not (e.g., Saint-Aubin and Poirier, 1999; Nairne and Kelley, 2004). Moreover, neuroimaging studies have shown that the retention of item memory during STM-tasks is associated with activation of phonological and semantic processing areas in the bilateral temporal lobes, whereas non-linguistic brain areas in the right intraparietal sulcus are activated when processing order information in STM (Majerus et al., 2006b, 2008a, 2010). Quite interestingly, in Experiment 1 we found that serial order STM and not item STM shows a substantial correlation with block design of the WISC-III (*r* = 0.38 vs. *r* = 0.06). Martinez Perez et al. (2012a) reported a similar result (*r* = 0.48 vs. *r* = 0.28) for a group of Kindergarten children. Taken together, the evidence seems to indicate that the partial independence of both STM processes is not attributable to a difference in phonological involvement, but is a result of the influence of non-verbal intelligence processes in serial order STM. Apparently, reconstructing the serial order of a number of elements is aided by active higher order restructuring of the material, possibly involving a visuo-spatial component mediated by the right intraparietal sulcus (Van Dijck et al., 2013; for a review see Majerus, 2009). Theoretically, we think that item STM could be considered as a necessary condition for serial order STM, but not as a sufficient condition, since it needs an additional non-verbal intelligence component. To conclude this issue, serial order STM, as it is involved with higher-order intelligent processes, seems not to be the right place to look for an explanation of a deficiency in the acquisition of the “modular” word reading process (Stanovich, 1988, 1990).

Our finding of an equal contribution of phonological processes in both STM tasks could be interpreted as support for the phonological deficit hypothesis of dyslexia as it indicates that verbal STM deficits (for item or order information) often reported in dyslexia can be explained by the poor phonological processing abilities that characterize dyslexia. However, our findings do not prove that serial order STM *per se* is phonological in nature. It remains to be seen in further empirical research whether the relationship between serial order STM and phonological abilities has to be attributed to serial order processing as such, or to the phonological nature of the stimulus material. As we adopted the serial order STM task from the study of Martinez Perez et al. (2013) we used the same phonological stimuli as they did. Although this task minimizes phonological demands, it would be worthwhile to investigate the role of phonological processes in serial order STM by using a task with non-phonological stimuli. In a study on Hebb learning (Staels and Van den Broeck, in press), we observed a substantial correlation between serial order learning of abstract visual forms that could not be verbalized with pseudoword reading and real word reading. Although this finding is suggestive for a role of serial order learning *per se* in reading ability, further research has to determine whether this relationship persists if item STM for visual abstract forms is tested. We suggest using a design in which item vs. serial order STM tasks, and phonological vs. non-phonological item material are bifactorially manipulated.

The main purpose of Experiment 2 was to investigate which STM process, item memory or serial order memory, is most closely related to orthographic learning, the process by which a beginning reader stores the orthographic details of specific words. As the ability to decode unfamiliar written words into their spoken equivalent is the central means by which orthographic representations are acquired, Martinez Perez et al. (2012a) suggested that STM for serial order information could also be important for the acquisition of orthographic representations. Again the results were unambiguous. Only item STM was significantly related to orthographic learning, and only in the most sensitive test of orthographic learning, i.e., orthographic choice (see Staels and Van den Broeck, 2013). Serial-order STM capacity, on the contrary, did not show any positive relationship at all with orthographic learning. Congruent with these findings is the robust observation in both experiments that word reading ability is more strongly related to item STM than to serial order STM. Again, more research is needed to find out whether the nature of the stimulus material (phonological or not) influences this relationship.

It is important to note that in Experiment 2 we tested a novel prediction made by Martinez Perez et al. (2012a) concerning the role of serial order STM in orthographic learning, but no attempt was made to replicate their longitudinal study. Hence, our differing conclusions about the role of serial order STM may stem from the fact that we measured orthographic learning and serial order STM concurrently in already literate children, while Martinez Perez et al. (2012a) measured serial order STM in kindergarten and followed up the children for their reading ability at the end of first grade. Although there is ample evidence that (serial) phonological recoding constitutes the first step in orthographic learning (Share, 1995, 2008), it is possible that the role of serial order STM in orthographic learning is less pronounced in literate children than in beginning readers, because with increasing reading ability orthographic learning may depend more on already existing orthographic structures. However, the results of the two studies are probably more in accordance than at first sight appears. In the study of Martinez Perez et al. (2012a) serial order STM was not a stronger unique predictor of later non-word reading after controlling for item STM than item STM was after controlling for serial order STM (equal beta's). Only after additionally controlling for phonological awareness, serial order STM predicted somewhat more unique variance in non-word reading than item STM did, although the difference in the beta's (0.31 vs. 0.22) was not statistically tested and the proportion of explained unique variance (8%) was rather small. Clearly, more convincing empirical evidence is needed to sustain the hypothesis that serial order STM plays a substantial role in explaining reading (dis)ability.

### Conflict of interest statement

The authors declare that the research was conducted in the absence of any commercial or financial relationships that could be construed as a potential conflict of interest.

## Footnotes

^1^Although STA as a matching design is conceptually very similar to the idea of controlling for item STM capacity in an ANCOVA, ANCOVA or hierarchical linear regression analysis are only a few data-analytical techniques that can be used to apply the broader concepts of STA (see Prince et al., 2012).

^2^We thank the authors for kindly providing their data for further analysis.

## Appendix A

Examples of items used in the Item short-term memory task.

     /g u k/

     /z i l/

     /r a k/

     /r i s/

     /s o t/

     /b i m/

     /k e p/
